# Exploration of key stakeholder views and experience of specialist schools’ food: a qualitative study

**DOI:** 10.1186/s12889-026-26758-x

**Published:** 2026-03-21

**Authors:** L. McSweeney, M. J. Andrew, A. R. Jones, I. Gordon, L. Pennington, J. V. Woodside, M. Craven, S. Spence

**Affiliations:** 1 Population Health Sciences Institute, Newcastle University, Newcastle upon Tyne, NE2 4HH UK; 2 Centre for Public Health, School of Medicine, Dentistry and Biomedical Sciences, Queen’s University, Belfast, BT12 6BJ UK; 3 Newcastle upon Tyne, UK

**Keywords:** Specialist schools, Special educational needs, School food, School food standards, Healthy eating, Free school meals

## Abstract

**Background:**

School food standards in England aim to support healthy diets for all pupils, including those with special educational needs and disabilities (SEND). While dietary advice is consistent across groups, SEND pupils may face unique challenges such as physical and sensory-related eating and drinking difficulties and higher rates of overweight. Nearly 45% of specialist school pupils qualify for Free School Meals (FSM). Despite the potential for school food to improve health outcomes, research on UK specialist school food provision remains limited.

**Methods:**

A qualitative study was conducted in four purposively sampled specialist schools in North East England. Data were collected via online and face-to-face focus groups and semi-structured interviews with key stakeholders, including pupils, parents/carers, Allied Health Professionals and school staff. Recruitment was supported by designated school contacts. Consent and assent were obtained using accessible formats. Discussions explored school food provision, dietary needs, and influencing factors. Sessions were recorded, transcribed, and analysed using thematic framework analysis. Data collection occurred between October 2024 and January 2025.

**Results:**

Twelve focus groups and eleven interviews were conducted across four specialist schools. Stakeholders highlighted efforts to meet pupils’ individual mealtime requirements, though challenges with modified diet provision and accommodating sensory preferences were common. Menu accessibility, food variety, and communication about food intake varied. Staff and families collaborated to support pupils, but concerns about hunger, food quality, and FSM uptake persisted. Some schools reported poor availability of foods suitable for modification. Variable flexibility in food ordering led to challenges in meeting individual pupil needs and concerns about dietary sufficiency in some settings. Stakeholder menu planning involvement differed across schools and catering models. Awareness of School Food Standards was mixed, with financial and staffing constraints affecting implementation.

**Conclusions:**

Specialist school food provision must accommodate diverse pupil needs and comply with School Food Standards despite resource constraints. Challenges in meeting individual pupil needs contribute to low uptake of FSM among eligible pupils with potential negative impact on the nutritional quality of FSM and on service viability. Future specialist school food interventions co-created with all stakeholders will ensure the feasibility and acceptability of interventions which aim to deliver equitable access to healthy school food for all pupils.

**Supplementary Information:**

The online version contains supplementary material available at 10.1186/s12889-026-26758-x.

## Background

A higher proportion of pupils attending UK specialist schools [1] are eligible for Free School Meals (FSM) compared to all school pupils (47% vs. 22.5%) [2], reflecting that UK children with special educational needs and disabilities (SEND) are more likely to grow up in poverty. FSM have the potential to improve pupils’ healthy eating; a school lunch provides a more nutritious meal for younger children in mainstream schools than a home-packed lunch [3]. However, according to O’Connell et al., only 1 in 4 FSM-eligible specialist school pupils are taking up their entitlement [4]. Pupils with SEND also have higher rates of overweight compared to same-age pupils without SEND (11.2% vs. 8.2% respectively) [5], making the provision of healthy school meals particularly important.

In England, school food standards [3, 6] apply to the food and drink on offer across the whole school day. Lunchtime regulations state the permitted frequency and availability of certain foods and drinks, for example, there should be no more than two deep fried food products per week, at least one portion of vegetables or fruit per day and no confectionery [7]. These standards apply to all maintained schools, including specialist schools.

Current dietary advice for pupils with SEND is the same as for pupils without SEND, though the energy requirements of children with some neurodevelopmental or neurological conditions associated with SEND e.g. low or high muscle tone may necessitate individual assessment [8].

Pupils with SEND may face specific challenges in relation to maintaining a healthy diet due to a range of factors including co-existing health conditions, medication, and/or physical and non-physical (sensory) eating and drinking difficulties [8]. Sensory eating difficulties relating to the appearance, texture, temperature or smell of food can lead to limited dietary repertoire and restrictive eating patterns [8]. Consumption of energy-dense, nutrient-poor processed foods (e.g. fries) and refusal of fresh foods (e.g. fruits and vegetables) are common [8]. Studies exploring UK specialist school food provision and stakeholder views are lacking [9].

## Methods

### Aim

To explore specialist school key stakeholders’ views and experiences of school food, their perceptions of healthy food provision, and factors influencing food choices.

### Study design and setting

Qualitative study comprising focus groups (FG) (online and face-to-face) and online one-to-one semi-structured interviews in specialist school settings (Table 2). Ethical approval was received from the Faculty of Medical Sciences Ethical Review Committee, Newcastle University.

### Schools

Four specialist schools in North East England were purposively sampled: one primary, one secondary, and two providing both primary and secondary education to include diversity of location (urban and rural), school catering (in-house, external, local authority (LA)), and percentage of free school meal eligibility (Table 2).

### Stakeholders’ recruitment, data collection and consent/assent

We spoke with each school Head Teacher (HT) prior to recruitment to provide an outline of the study and the requirements for taking part. In each school, a HT appointed liaison person helped with the recruitment of staff, parents and children and the organisation of meeting times etc. The designated contact disseminated study information to parent/carers and school-based stakeholders (including allied health professionals) and shared consenting participants’ contact details with research associates LM and ARJ. Caterer providers were directly contacted by ARJ and invited to participate in an interview. Adult stakeholders (Table 1) were sent the study participant information sheet and completed an online consent form using Microsoft Forms. Parent/carers of participating pupils were sent an Easy Read participant information sheet to share with their child. Parent/carer consent was obtained for participating pupils via Microsoft Forms. All participating pupils provided written assent.

**Table 1:** Method of data collection and consent/assent for each stakeholder

Stakeholder	Data collection method	Method of consent/assent
Young people (aged 11–18 years)	Face-to-face focus group	Parental online and in-person pupil assent
Parents/carers	Online focus group	Online
Head teachers/ school governors	One-to-one online/telephone interview	Online
Teaching staff	Online focus group*	Online and in-person
Allied health professionals (AHP) e.g. dietician, occupational therapist etc.	Online focus group	Online
Catering staff/ chefs	One-to-one online/ telephone interview	Online
Caterer provider	One-to-one online/ telephone interview	Online

*Two schools requested a face-to-face focus group for teaching staff

### Data collection

Stakeholders who consented to participate were sent a Teams invite to join the online FG/interviews; online methods were practicable for inclusiveness and convenience of stakeholders and logistics for staff. Stakeholder-specific topic guides were used to facilitate discussions (Appendix 1). Given the exploratory nature of the study and the novelty of the research area, topic guides were developed iteratively, drawing on the limited available literature, the team’s expertise in school food and SEND health, and discussions to ensure topic guides/questions would capture a wide range of issues relevant to participants. Questions focussed on areas such as school food provision, pupil’s experience of the dining hall environment, development and access to menus, and catering for those with a modified diet or specific dietary requirements, including sensory needs. The focus groups and interviews were video or audio-recorded with the participants’ consent and transcribed verbatim using a Newcastle University approved transcribing company. The FGs/interview length ranged between 20 and 60 min. Following transcription, recordings were deleted. One pupil did not agree to be recorded, therefore, for that focus group, detailed notes were taken. Data collection took place between October 2024 and January 2025. Participating schools received a £500 donation, and parent/carers and pupils each received a £50 shopping voucher.

### Data analysis

Transcripts were loaded into NVivo 14 [10] and analysed using inductive thematic framework analysis [11], to identify, describe, and interpret key patterns within and across stakeholders and themes within the phenomenon of interest [12]. An inductive approach allows themes to develop organically from the data rather than being predetermined by existing theories. This bottom-up method is especially useful for investigating areas with limited prior research, as it reflects genuine participant perspectives and minimises bias introduced by theoretical frameworks [13].

Data were analysed by the qualitative researcher who led on the stakeholder FG/interview (LM, ARJ, IG) all three researchers adopt a qualitative epistemological stance and bring extensive training and experience in qualitative research methodologies. Their positionality is informed by prior professional engagement with schools, which shapes their understanding of educational contexts and interactions.

LM reviewed the combined findings to identify overall themes, with specific pertinent highlights from each stakeholder group. Due to the volume of rich data collected, some stakeholder quotes are shared as supplementary material (Appendix 2).

## Results

Twelve focus groups: three pupil, four parent/carer, four teaching staff, one combined Allied Health Professionals (AHPs) and eleven individual interviews were completed across the four schools. Table 2 outlines the number of participants per school/stakeholder group, school type, location, age of pupil provision, percentage of pupils receiving FSM and catering provider type.

**Table 2:** School characteristics, (location, age of pupil provision, % of pupils eligible for Free School Meals, catering provider and number of stakeholders participating in focus groups or interviews

School	School A	School B	School C	School D
Location	Semi-rural	Urban	Rural	Urban
*Age of pupil provision	Nursery, secondary & sixth form	Nursery & primary	Nursery, secondary & sixth form	Secondary & sixth form
% of pupils eligible for Free School Meals	59.8	54.9	48.5	72.0
Catering type	In-house catering provider	LA catering provider	External catering provider	LA catering provider
Participants (n)				
Young people	5	N/A	4	5
Parent/carers	3	2	2	3
Head teacher/ Governor	2	1	1	1
Teaching staff	8	5	3	3
Allied Health Professionals	5 across three schools			
Catering Provider	N/A	2	0	2
Catering Manager	1	0	0	0
Catering staff/on-site chef	0	1	1	1

*Age of UK specialist school provision: Nursery – 3–4 years; Primary – 4–11 years; Secondary – 11–18 or 19 years; Sixth form college – 17–18 or 19 years (note not all schools have a sixth form college). Some specialist schools extend their provision to support students through post18 education

School A provided an in-house catering service, the catering manager was part of the catering team and so for the purpose of analysis was categorised as both. Schools B and D had the same LA catering provider, therefore the two catering providers interviewed represented both schools. School C used an external catering provider, however, despite several attempts, no interview was completed. All schools cooked meals on-site.

In total, eight main themes and 38 sub-themes were identified (Table 3). Illustrative theme quotes are labelled by stakeholder and school i.e. (PL) pupils; P (parent/carer); HT (head teacher/governor); TS (teaching staff); AHP (Allied Health Professional); CP (catering provider); CM (catering manager); CS (onsite catering staff/ chef); and schools A, B, C, D.

**Table 3:** Themes and sub-themes from focus groups and interviews

Theme	Sub-themes
Meeting and supporting pupil’s individual needs	Knowledge of pupil’s needs Support for pupils to choose and eat Restrictive eating Modified diets Flexibility around food Portion sizes Cultural and religious requirements Hunger
Food choice, decision makers and provision	Food and drink availability Food and drink accessibility Catering staff budget constraints Healthy food provision Packed lunches/school lunches Other food eaten at school Food rules
Dining hall environment and procedures	Dining hall atmosphere Alternative provision to dining hall Staff support over lunchtime Flexible lunch arrangements
Communication	Pupil voice Parent/carer voice Feedback from school Family, school and caterers working together
Menu planning and communication	Development of menus Council use of mainstream menus Input into menu planning Parental access to menus Pupil’s access to menus
Impact of SEND on pupil’s food consumption	Lack of interoception Sensory considerations Concerns about over-eating Concerns about underweight Impact of pupil’s home life
School food standards, guidelines and policies	Awareness of school food standards Awareness of school food policies Challenges with implementation
Development of pupil’s food life skills (supplementary material, appendix 2)).	Food and food preparation as part of the curriculum Encouragement to try new foods/tastes Socialisation and eating skills

### Meeting and supporting pupil’s individual needs

Overall, parent/carers reported that support for their child’s specific health and/or educational needs was good:

*“They’re [school] very good with [sic] when you speak to them about concerns*,* they’re very good with trying to meet those concerns” (P*,* school B).*

Teaching staff described how they got to know the individual pupils and their preferences, including restrictive or sensory practices. School staff worked closely with families and health professionals to meet specific dietary requirements:

*“…but if we’ve got those restricted eaters*,* I think we just go with parental choice and act on the advice of SALT (speech and language therapist) and the paediatricians and things to make sure we’re putting the right stuff in front of them [pupils] and in them” (TS*,* school B).*

Pupil’s dietary choices were respected and supported in all schools. For pupils with restricted diets or sensory preferences, one Head Teacher described how support to try new tastes and foods was provided:

*“…for us those faddy eaters*,* it’s trying to understand the relationship they have with food and the sensory implications …So… if a child likes crunchy crisps*,* then are there things that we could make crunchy…Just trying to think of the ways we can get our young people to accept other foods*,* [HT*,* school A].*

The pupils in school A pre-selected their food via online menus in the classrooms, however catering staff allowed pupils to change their mind if the food collected at the serving hatch was not as expected as stated by one pupil:

*“Every day*,* you know that*,* if you don’t like one thing*,* you don’t have to…” (FG facilitator*,* school A)*.

*“You can have something else” (PL*,* school A).*

For many, pre-choosing a meal was too challenging, and teaching staff described how most pupils chose their lunch at the serving hatch or using a visual menu:

*“But I would say the majority of people go up and see what’s at the hatch” (TS*,* school D).*

Pupils in school D stated that having the ‘healthy options’ highlighted on the menu would help them to make healthier choices:

*“Write the healthy things on the menu separately” (PL*,* school D).*

All schools offered pupils a range of foods, including healthy options. However, as stated by one parent, not all schools offered a choice of meal to pupils on a modified diet:

*“My son has to eat food that’s blended [modified]. He gets no choice over what he gets for his lunch. It’s just one meal for every single person who is on that diet…I kind of get the feeling it’s just whatever they’ve got left over. They’ll just stick that in the blender and whizz it up*,*” (P*,* school D).*

The challenges associated with modified diet provision were raised across all stakeholders in three schools. AHPs and staff reported how CS could struggle to provide modified diets within the resources available:

*“I think the food*,* for the children… who need modified diets*,* is often quite limited…in terms of the children who can manage more texture than that*,* so like the soft and bite-size*,* that’s really tricky for the kitchen to get right” (AHP).*

Teaching staff also reported the limitations faced by CS:

*“There are a lot of children. When you think how many [chef is] covering*,* and [they’ve] got all the different blends and minced and moist and all of those…[they’ve] got an awful lot on their hands…[they] do very well because*,* I mean*,* I think [their] budget’s pretty terrible” (TS*,* school B).*

Whilst it was acknowledged that the chef/CS were working with very limited resources and a lack of modifiable ingredients, some pupils had no choice in what they received. In one school the meal components were always blended together, rather than kept as separate components, which staff described as unappetising.

Staff were also concerned that some pupils may not receive adequate nutrition or remain hungry after meals due to small portion sizes. Parent/carers also described small portion sizes:

*“I agree… that the portions aren’t necessarily big enough. [child] always comes home absolutely starving*,* we’re having to send things in for him to eat in the middle of the afternoon because he doesn’t get to the end of the day without being hungry” (P*,* school D).*

In school D, the pupils spoke about feeling hungry in the afternoon:

*“So sometimes*,* once you’ve finished your meal deal*,* you might still be hungry”? (Facilitator*,* school D)*

*“Yeah” (PL*,* school D)*.

*“Yeah*,* your belly rumbles too much*,* when you’re working” (PL 2*,* school D).*

Moreover, AHPs reported that pupils with sensory needs who are used to eating a certain cultural diet at home could struggle with the vegetarian options offered to those following, for example, a halal diet:

“*We’ve got children who will eat well at home because it’s rice and because of the way they eat and what they eat…It’s more like curries*,* and rice*,* and meats and things cooked in very different ways. Then they come in and they’re being offered a Quorn fillet*,* and wedges and sweet corn. It just is so alien to them that it’s really hard to get them*,* especially if they do have some of the sensory nuances that we’ve got” (AHP).*

### Food choice, decision makers and provision

Food and drink availability and perceived healthiness varied depending on the school context (in-house or LA catered). School staff reported that breakfast and lunch provision included healthy options:

*“But I think we have healthy breakfast. The dinners are balanced*,* and then we’ve got fruit and then the water and juices” (TS*,* school C).*

Food provision out with mealtimes e.g. snacks varied between schools and sometimes between classes. Two schools provided fruit for pupils during the day. Some pupils also had access to toast or biscuits in classrooms. As described by teaching staff, parent/carers often sent snacks with their child, especially if the child had a restricted diet:

*“There’s no snacks provided by the school. There is a biscuit in a double lesson…but snacks at break time are usually provided by the parents” (TS*,* school D).*

Staff were aware that some snacks provided by parent/carers may not be healthy:

*“I think a lot of the parents do send in snacks as well*,* sometimes they can be unhealthy because parents are sending it in…you don’t want to waste the food” (TS*,* school B).*

AHP described the limited provision of snacks that could be safely modified which often led to some pupils only receiving the main meal or having an unhealthy processed option.

*“They ask parents to send in [snacks]*,* and a lot of the parents don’t*,* for whatever reason that may be financially [sic] or their pressures. So*,* often I find the children who*,* especially*,* have a puree or a Level 5 diet [Minced and Moist* [14]*]*,* their range of snacks within the school day is probably not that healthy” (AHP).*

Some schools issued guidelines about the types of foods that could be brought in from home. These were not always adhered to as parent/carers and staff were most concerned that ‘something’ was eaten, irrespective of nutritional value:

*“We’re [school] not allowed fizzy drinks and energy drinks*,* and it’s usually*,* it’s not sweets and things. But with an understanding of somebody might not- like*,* student preference as well. So*,* you’re not going to go and grab something off someone because it doesn’t fit the healthy box*,* but it might be the only thing that student would eat” (TS*,* school D).*

*“We’re always reticent to give guidance in that sense [packed lunch contents] …we will speak to parents when we do annual reviews…but it’s really difficult if you put anything else in front of the child*,* and they refuse to eat.” (HT*,* school C).*

Concerns were expressed by caterer providers about the lack of uptake of FSM by eligible pupils:

*“You want to make sure that the children that are on free school meals are fed*,* so that then becomes an issue as well…as a caterer*,* obviously*,* we want the biggest free school meal uptake and paid meal uptake*,* because otherwise*,* it’s not worth running a service. So then*,* it all becomes monetary and budgetary as well. So*,* we have all of those challenges to deal with*,* because if we don’t provide a menu that the children are happy with*,* they go on packed lunch.” (CP*,* schools B and D).*

Some pupils with specific sensory preferences were also at risk of receiving a limited diet. Many of the pupils were reported to bring a home-packed lunch due to restrictive eating and/or sensory considerations as described by one AHP:

*“Most of our sensory [pupils] will bring in*,* yeah. Even if they were*,* maybe*,* entitled to a free school meal*,* they’re having to bring in a packed lunch” (AHP).*

Schools mostly accepted parent/carers sending in food from home; however, it meant some pupils ate the same foods daily or had unhealthy options. Stakeholders, including some pupils, reported that a home-packed lunch was the preferred option for many (including those eligible for FSM) who had specific food preferences. Parent/carers and staff felt reassured that ‘something’ was being eaten, albeit not the healthiest option.

### Dining hall environment and procedures

Both the teaching staff and the pupils agreed that the dining hall could be noisy and overwhelming:

*“It gets us stressed a bit” (PL*,* school A)*.

All schools provided flexibility by having alternative eating spaces for those who found the dining hall overwhelming:

*“That’s always our long-term aim to get young people into the dining hall but the majority of primary classes their ASD (autism spectrum disorder) is such that they need to eat in their classroom so it’s not overwhelming” (TS*,* school A).*

Some teaching staff struggled to meet the specific mealtime requirements of their pupils within an allocated dining hall slot. AHPs noted that lunchtime was more relaxed if eaten in the classroom as there was less time pressure:

*“I think*,* moving from the dining hall into the classrooms*,* for some of the children*,* has given them more time. So*,* what you would have found in that dining hall was that the dinner ladies were finishing their shift*,* it all needed to be cleaned up…A child’s independence doesn’t stop when on a time constraint of somebody else” (AHP).*

Pupils eating in alternative spaces still received a choice of what to eat and continued to eat with peers. In one school, the set-up of the dining hall did not meet the needs of some pupils:

*“It is very noisy. And also*,* because I know it’s round tables*,* but when you have wheelchair users try to get out*,* yeah*,* it’s quite difficult to navigate” (TS*,* school D).*

Some pupils spoke about the busyness of the dining hall environment and not having time to decide what to eat at the serving hatch:

*“It’s hard for me to pick*,* because half of the time- like*,* because [chef] is always busy” (PL*,* school D).*

Teaching staff from three of the schools reported that staff availability to support lunchtime could be challenging:

*“I think it’s hard when you’ve got a few children in class that have feeding plans that need quite specific support…it can make it quite hard to support some of the others that maybe just need those life skills of cutting or talking about their food…or*,* yeah*,* table manners” (TS*,* school B).*

### Communication

All schools actively communicated with families to ensure that pupils’ needs were considered. There were many examples of staff and families working together to resolve individual pupil issues:

*“We’ve got a young girl who’s got a…. compulsion to eat. We work with home to make sure that she’s only having one dessert a week” (TS*,* school A).*

The schools used various methods to communicate with parent/carers, these included regular telephone calls, face-to-face at drop-off/pick up, written daily diaries and online message boards. Some parent/carers received daily feedback of what their child had eaten that day; however, this was inconsistent across schools beyond a certain age as described by one parent:

*“When she [child] was in reception*,* they [school] used to send home daily diaries which used to be a brief description of what they’d done and what they’d eaten…which for us*,* with her being non-verbal*,* was useful. But when they move up the years*,* they stop the daily diaries… that’s difficult because she’s not going to come home and tell us what she’s had” (P*,* school B).*

Whilst parent/carers appreciated the information they received about their child’s food intake they reported this could be improved:

*“Occasionally*,* I’ll get*,* “Oh*,* he has had such-and-such for dinner*,*” but they don’t tell you if he ate it all*,* or if he ate half of it*,* or actually if he enjoyed it. They’re just very brief*,* so I feel communication around lunch could be better” (P*,* school D).*

Parent/carers were clear that they wanted daily communication about what their child had eaten, especially if they were unable to communicate what they had eaten, underweight, or had other specific needs. Parent/carers suggestions to improve communication were made:

*“…we have this app*,* don’t we? Dojo. Can they [school] not add something to that or do a separate one where you quickly just tap in to make it as quick… what they’ve had and how much they’ve ate [sic]?” (P*,* school C).*

Conversely, some staff reported that only the minority of parent/carers wanted feedback about food consumption:

*“…so we have some of our young people in class whose parents have requested that on a daily basis and so that is just done by… rather than it being a phone call which can sometimes take longer*,* it’s just a quick email that I would send in terms of variety and the quantity that’s been eaten…I feel that that’s something that a lot of ours [parents] are not as interested in now*,* it is just for the few” (TS*,* school A).*

The pupil voice was also emphasised as important by teaching staff:

*“I think that’s one of the good things…because they’ve [pupils] got a voice about what they can eat and you don’t see so many unregulated times at lunchtime because [chef] will really listen to them and they feel listened to (TS*,* school A).*

### Menu planning and communication

Menu planning and cycles were discussed by several stakeholders; a 3-weekly menu cycle which changed twice a year was common. Due to differing catering provision models across schools, the menu development process, individuals involved, and flexibility varied. For example, in School A (in-house model), the catering manager led menu development. Meals were deconstructed to component parts allowing pupils to choose what they wanted:

*“We have a core three-week menu*,* and it cycles… but everything is deconstructed…it’s all totally separate*,* like you would go into a buffet*,* it’s done like that…some of those students will have chicken and gravy*,* because they don’t like the vegetables…the other alternative to that yesterday was a tomato and basil pasta…some of them wanted the tomato sauce over the chicken and the rice. .so*,* they can have itemised meals that way.’ (CM*,* school A).*

In school A, pupils had input into menu planning; the catering manager altered the menu according to their advice:

*“The student council …have input into the menus as well…so*,* we give them two options*,* and then they pick” (CM*,* school A).*

In school C, the menu was developed by the external catering company and then adapted in-house to meet pupils’ needs. As explained by one Head Teacher, a greater number of individuals were involved in this process:

*“We use an external catering company. So*,* we work with them in terms of menu choices and making sure they’re varied…we do have input as well from dieticians to certain children*,* and meals*,* and diets*,* as well. We take that on board*,* we follow their plans and protocols…. individual class teachers will have input… Obviously*,* the school cook… parents. We work with parents as well” (HT*,* school C).*

Schools B and D both had an LA CP. The CP described the process of menu development and who is consulted. It was acknowledged that menu adaptions are made by the on-site kitchen team to meet specific needs:

*“It’s all in a menu planning computer. And I just have to make sure that I balance my menus wherever possible*,* make sure that I’m getting the fruit in every single day…We always have potatoes available*,* veg…and their protein every day… like always have mash on*,* always have gravy on…and…regardless of what we have on that day*,* we always have custard on*,* and it’s for the children who always want custard…that is in the special school…who we’re dealing with all the dysphagia levels*,* and their acute dietary needs” [CP schools B and D).*

Some stakeholders, such as one Head Teacher, suggested that they had no or limited say in menu planning and would like to have more input:

*“We go off the primary menu [mainstream school]…the cooks and chef*,* they’ll be working from the set menu*,* what they’re told to provide…I don’t think there’s any [teacher input]*,* to be honest… the SALT team will look at what’s available and inform the kitchen staff of how that could be presented according to the child’s feeding plan…In terms of what’s provided… all schools have got the same menu*,* so it’s not really a school-based thing…*,* where we could actually respond to people’s thoughts and change things. It’s because it’s external” (HT*,* school D).*

Many parent/carers described a lack of consultation about the menus and most had no access to a menu. Some were aware of the menu cycle and had received this at the beginning of term. One school posted the menu on its social media page. Parent/carers of children with specific needs were often sent a menu so that they could discuss with staff what their child might eat:

*“I personally got a printout. And they [school] would send me one at the start of every term…and then they would just ask me to feedback on which dishes [child] would like. Because he isn’t verbal to make that selection*,* you know…but I know he does try other stuff that I haven’t selected*,* so*,* that’s great (P*,* school A).*

Pupil access to menus differed between schools. Some schools displayed the menu in the classroom or used online drop-down menus so that pupils could choose what they wanted in advance:

*“Because we have*,* like*,* a huge board in our class*,* it’s like an electrical board. And it’s just names and what they’ve picked. It’s like a tally chart*,* a bit. It will say ‘Monday’ and then you pick. It includes dessert too. And then it goes*,* next one*,* ‘Tuesday’*,* …” (PL*,* school A).*

However, staff in school D described poor menu accessibility for pupils:

*“[The menu is] not accessible in any way to our students. It’s very complicated because it runs on a three-week cycle. So*,* you’ve got to look at the tiny dates on the side to work out which week you’re in. And then*,* the photos don’t match what you’re getting on your plate” (TS*,* School D).*

### Impact of SEND on pupil’s food consumption

Individual pupil needs could impact their ability to consume certain foods. The AHPs and some teaching staff described how a lack of interoception affected some pupil’s ability to discern hunger, satiety, or thirst:

*“When somebody is trying… “It’s time to have a drink*,*” or “Have a drink*,*” they’re [pupils] just very resistant because they just don’t feel thirsty or hungry” (AHP).*

*“It’s knowing what’s hunger and what’s lack of awareness of being full. Some of them [pupils] would ask for food all the time” (TS*,* school B).*

AHPs recognised the importance of mealtime participation, acknowledging a trade-off sometimes between taking part and eating healthy food:

*“The choices that some of our*,* particularly*,* sensory-challenged children have*,* which wouldn’t necessarily be considered healthy choices. (Laughter) You want to have those because you want them to be able to chew*,* take part in eating*,* along with their peers*,* but you wouldn’t necessarily want to have them on the menu” (AHP).*

CS described the differences between catering for mainstream and specialist schools:

*“We do have a little bit of lenience here. We’ve got to put gravy on every day*,* because a lot of the children will need their food wet. Again*,* we’ve got custard on every day…In my other primary [mainstream] school we wouldn’t necessarily have gravy and custard on every day” (CS*,* school C).*

Having a restricted diet raised concerns about some pupils being underweight and/or hungry. Parent/carers were particularly anxious about their child eating enough:

*“My son’s very thin as well but he does eat. It is always a concern. We need to know what they’re having*,* how much and if it’s safe” (P*,* school C).*

Teaching staff work with the pupils and families to try and address weight-loss concerns:

*“There’s a little boy who has lost a lot of weight and is quite poorly and really restrictive and the only thing he would eat was pizza so [chef] made him a whole pizza to himself*,* put in the freezer and that he got every day while we were trying to get his weight up” (TS*,* school B).*

Despite best efforts to use a flexible approach to school food to meet pupils’ needs, one Head Teacher highlighted that this did not always result in a healthy, balanced diet:

*‘I would say*,* it meets the needs of students with SEND well*,* in the sense that we individualise what we deliver for students. I think the issue with that is that you’re catering very much to the student’s demands*,* and not necessarily what would be constituted as a totally balanced and healthy diet” (HT*,* school C).*

Parent/carers and staff were also aware of over-eating practices and the excessive consumption of refined carbohydrate foods as one parent described:

*“He’s [child] got an underactive thyroid*,* so it’s quite hard to keep his weight under control anyway…now we don’t give him breakfast at home*,* he goes in and likes having that bagel [breakfast club]” (P*,* school A).*

Some teaching staff spoke about how a pupil’s home life may influence their food choices and about how their behaviour at home may influence the food that is provided, healthy or not:

*“I think as well*,* most of it is our parents have a really hard life when they’re at home. I think food can be a moment of relief from that. And if the kid wants to eat chicken nuggets…if it’s a fight to give them something*,* if it’s going to result in a full crisis where the kids hurting himself or trying to hurt them or trashing the house or whatever it is… It’s very easy*,* as professionals*,* to go*,* “You should try that” (TS*,* school C).*

### School food standards, guidelines and policies

Stakeholders were asked about their awareness and knowledge of school food policies and standards, including the School Food Standards [6] or local school healthy eating policies. HT and TS knowledge of the School Food Standards tended to be general and vague. However, the Governor at School A thought that their school exceeded the School Food Standards through in-house catering:

*“Within [school name with in-house catering] the chef works within the government guidance for school meals. I think we probably exceed that*,* in terms of the amount of freshly bought local produce and things like that*,* but also in terms of offering vegetarian or vegan options within the daily offer” (Governor*,* school A).*

AHPs had some understanding of what the guidelines entailed:

*“I’ve heard of it*,* probably*,* more in relation to mainstream schools*,* but I’m sure it would apply against both*,* but in terms of getting a certain proportion of your calories in per day and having one to two portions of fish a week and things*,* that the government sets out that the schools should try and stick to” (AHP).*

Most parent/carers did not know exactly what guidelines related to, but could describe certain aspects:

*“It’s decent quality and quantity of food for the children” (parent*,* school B).*

Parent/carers in school D were aware their school had a healthy eating policy, but this was noted to be out-of-date and not fit for purpose as displayed on the website:

*“It definitely isn’t practised*,* I think. It’s like a lot of things: anyone can have a policy*,* but it’s definitely not what’s happening at school. You just have to take one look at their menu and go*,* “Yeah*,* right” (parent*,* school D).*

Some staff associated the School Food Standards with health and hygiene regulations. Discussions with the CS/caterers were more detailed, only one CS participant was unaware of the School Food Standards. One member of the catering staff provided examples of guidance they were required to follow in terms of food items and portion sizes, and discussed the flexibility/leniency allowed in relation to the School Food Standards within the Specialist School Setting:

*“Well*,* it’s like the graph…and you know how much carbs to give them. And the vegetables*,* and how they should be portioned out… that’s all done within the menus that we provide that come through the nutritionist…I was in a mainstream primary school before here*,* and it was still the same standards of everything and all the criteria that you would meet… we’re just a little bit more lenient here*,* because sometimes they do struggle with the foods” (CS*,* school C).*

The implementation of standards and policy was highlighted as a complex task. The LA CP spoke about internationally recognised levels of thickness of foods and drinks for people with dysphagia (swallowing difficulty). School staff highlighted a lack of training on this, and had undertaken self-directed learning:

*“So*,* the dysphagia levels… as in*,* how pureed or soft the meals can be*,* for swallowing…they’ve [staff] had technically no training*,* like official training*,* when I do the menus*,* I actually put on what levels each of the dishes can be done on dysphagia… then*,* it helps [them] know what choices they’ve got for the menu as well…it was something that I’ve*,* kind of*,* had to work out for myself” (CP*,* schools B and D).*

Food and staffing costs influenced School Foods Standards implementation. A CP described the conflict of financial restraints from a provider perspective. The availability of staff with the skills to prepare school meals adherent to the School Food Standards could be limiting:

*“Challenges are things like budgets*,* skill sets of staff*,* because school food standards will maybe have dishes that you have to create that involve a skill. Trying to get that many skilled chefs these days is very hard to get in school meals. It doesn’t pay strong enough to get a good level of cooking standards*,* so that’s always a challenge” (CP*,* schools B and D).*

## Discussion

This study is one of the first to report the views of multiple stakeholders on specialist school food provision. The findings identify multiple inter-related factors which influence the food choices made by pupils in specialist schools, with subsequent impact on FSM uptake.

Several of the issues discussed by stakeholders are also relevant to mainstream settings. Challenges with the variety, quality and familiarity of foods offered, parental concerns about the nutritional quality of school food, or a desire to provide familiar and preferred foods from home, challenges with dining hall environments, staff resource to support mealtimes, and reduced FSM uptake are common to mainstream settings [15–21]. Despite some challenges being common to mainstream and specialist school settings, there is nuance specific to specialist school settings relevant to the impacts on pupils with SEND [18, 22], who are more at risk of under- and over-nutrition. Aspects such as, the provision of appealing, nutritionally complete meals for pupils receiving a modified diet; menu accessibility issues; conflicts between the need to provide healthy foods compliant with School Food Standards versus preferred and often less healthy foods to avoid hunger or undernutrition, and the need for multiprofessional and broader stakeholder input to menu planning are specific to specialist school settings.

Although pupils’ food preferences contribute to school food choices and uptake in mainstream settings [17, 19, 20], stakeholders in all participating specialist schools described food preferences driven by pupils’ sensory needs and/or restrictive diet as being key to school food choices, consumption and school meal uptake. School caterer provider may also have an influence. Schools adopting an in-house catering model appeared to have greater autonomy to order foods more appropriate to varied and specific pupil requirements, whereas others highlighted challenges with meeting individual pupil needs within the foods supplied.

Restrictive diets creating a reliance on safe, preferred foods resulted in families of specialist school pupils providing a packed lunch containing foods their child would eat, even if these preferred foods were less healthy. Some families also mentioned they provided preferred snacks or condiments to encourage adequate intake. An additional challenge for specialist schools is the need to deliver an appealing nutritionally varied and complete diet that meets the needs of pupils on a modified diet. Dining hall noise and queues are challenges reported in mainstream school settings [16, 19]. For specialist school pupils, the acceptability and tolerability of the dining hall environment is affected by pupils’ sensory needs. In all specialist schools included in this study, alternative eating spaces were provided to help pupils manage mealtimes e.g. option to eat in classrooms, which further impacts on mealtime staffing resource.

Parent/carer and pupil access to menus was mixed. Whilst variable parent access to menus is not unique to specialist school settings; variable provision of menus in a format accessible to pupils with SEND affected some pupil’s opportunity to select their meal in advance. Staff in some schools reported that choosing a meal at the dining hatch was easier for pupils, though requires adequate staff support in the dining hall and may not allow sufficient time to consider the healthiness of foods on offer leading to rushed choices and reliance on familiar preferred foods.

The need for more specialist input to menu planning from all stakeholders is unique to specialist school settings. The level of input varied according to catering provider. Only one school discussed pupils helping with menu planning, overall, pupils had very limited or no input into menu development. Parent/carers also mentioned they wanted involvement in menu planning to increase the availability of foods suitable for their child Some stakeholders were aware that the School Food Standards provided guidelines for the types of foods to be served in schools to ensure a nutritional balance. It was acknowledged that the School Food Standards and the practicalities of feeding pupils with SEND did not always fully align. Specialist school CS described additional budgeting restraints and a limited pool of CS with experience of working within a specialist school setting as barriers to implementing the School Food Standards. The current lack of, and need for, formal training for staff to ensure that the safety and nutritional needs of all pupils can be met was highlighted.

Many CPs described the complexity of financial restraints for their setting, and the need to maximise the number of children taking up FSM to ensure the sustainability of a service capable of meeting diverse specialist school food requirements [4]. The provision of packed lunches comprising preferred foods may reduce FSM uptake, as well as potentially limiting opportunities to extend the repertoire of foods tried. Whilst similar challenges with FSM uptake are reported in mainstream schools [23], reduced uptake of FSM as found by O’Connell et al. [24] may further limit financial resource to provide sufficient variety to meet the needs of SEND pupils.

## Strengths and limitations

This is one of the first studies to collate stakeholder views of specialist school food provision and highlights several key opportunities to support healthier food choices for specialist school pupils. All stakeholders involved in specialist school food, including parent/carers, young people, catering, education, and health staff were included. The participating schools were selected to ensure diversity of age-group, location, and catering models used. Limitations include purposive sampling of participants. Parent/carers may have been motivated to do so because of their own or their child’s experiences of specialist school food. Their views may not be representative of a broader parent/carer group. Pupil participants were selected by school staff and could all express their views verbally without assistance from staff or technologies. Their views may not be representative of all pupils attending specialist schools. Additionally, we were unable to secure an interview with one of the school caterer/providers. All schools were in the North East of England, with a limited number of schools participating, therefore, findings cannot be generalised. No dietary data e.g., from menu analysis or dining hall observation were obtained to support qualitative findings.

## Implication for practice and policy

This study provides data obtained from four specialist schools in one geographic area of England, and so caution must be exerted in making recommendations for policy and practice. However, the study highlights several areas for further development work, and opportunities for practice change. Issues with menu accessibility were perceived to limit decision-making in advance of mealtimes, creating the need for higher levels of staffing support at mealtimes. The use of accessible menus as standard practice could support pupil autonomy in healthy food selection.

Limited choice for pupils on a modified diet or with restrictive diets potentially leads to inequitable food provision, particularly for pupils eligible for FSM, who may resort to bringing in modified food and snacks from home. The involvement of school staff, AHPs, and parent/carers in menu planning could help ensure availability of a broader range of foods suitable for modification. From a catering perspective, menu development requires consideration of strategies that reconcile pupil needs with statutory nutritional standards and budgetary restrictions to ensure equitable school food provision for all specialist school pupils. Finally, the development of school staff training resources on physical eating and swallowing difficulties would address described knowledge gaps and support safe mealtimes for affected pupils.

## Conclusions and future research

Those providing food for pupils attending specialist schools have the complex task of balancing pupils’ diverse needs with School Food Standards, health-related food requirements and budgetary restrictions to maintain a viable school food service.

There was concern over FSM eligibility versus uptake due to the high number of FSM eligible pupils bringing a packed lunch. Addressing the barriers to FSM uptake is key to ensuring the ongoing viability of the service and to ensure that pupils most in need have access to a healthy balanced meal in school.

Future research should also define the components of healthy specialist school food systems, including food provision, eating environments appropriate for pupils with SEND, and communication systems. Additional work should be undertaken to better understand how routine behaviours may contribute to current specialist school food practice and identify opportunities for positive behaviour change. The identification of appropriate meaningful outcomes for future interventions is also important, including outcomes relevant to health and healthy weight. Meaningful stakeholder engagement is required to facilitate the co-creation of appropriate and acceptable interventions to ensure equitable access to healthy school food for all specialist school pupils.

## Supplementary Information

Supplementary Material 1.

## Data Availability

The datasets used and/or analysed during the current study are available from the corresponding author on reasonable request.

## Abbreviations

SEND: Special educational needs and disabilities
FSM: Free School Meals
FG: Focus groups
AHP: Allied Health Professionals
HT: Head Teachers
CS: Catering staff
CP: Catering provider
LA: Local authority
