# Supplementary material for: Exploration of key stakeholder views and experience of specialist schools’ food: a qualitative study
Source: BMC Public Health. 2026 Mar 21;26:1398. doi: 10.1186/s12889-026-26758-x (PMC13126920; doi:10.1186/s12889-026-26758-x)
Supplement: Supplementary file 1 — Supplementary Material 1. [file 12889_2026_26758_MOESM1_ESM.docx]

**Appendix 1:**

**Topic guides**

**The CHOICE Study**

**Health professionals’ topic guide**

**Introduction**

- Thank participants for taking part and giving up time.
- Confirm they have read the participant information sheet and ask if they have any questions about the study.
- State the discussion will take around 1 to 2 hours and they are free to stop taking part at any time if they choose to do so.
- Confirm they are happy to go ahead with the discussion. Check they have completed the e-consent form, if not, ask for verbal consent and request the e-consent be completed post-interview.
- Inform participants that there are no right or wrong answers but that you just want to talk about their experiences of the school food and drink provision and process at their child’s school.
- Remind participants to speak one at a time and to respect other people’s views if they do not agree with their own.
- Confirm use of Zoom/Teams recording and they are happy to be recorded.
- *Remember to switch on the recorder*

**Questions**

1. Please could you introduce yourself, state your professional role, and the age range of child or young person you work with.

1. We want to find out what you think about the food served at school. How well does it meet the needs of students with SEND?

1. How does the mealtime environment in your school contribute to the food and drink choices made by pupils in school?

1. Dining hall set-up
2. Number of children eating at one time
3. Noise levels, other distractions
4. Placement
5. How are children supported at mealtimes

1. What other factors influence CYPs school food and drinks choices?

1. Do you think that the types of food and drink on offer are healthy, should these be changed in any way?

1. How well do the foods and drinks on offer cater for the needs of CYP with diverse eating and drinking requirements?
2. What provision is there for cultural/religious differences?

1. As health professionals, how much input do you have in menu development, including the range of food and drink options available? How often?

1. What level of input do you have in helping catering staff to appropriately adapt provided meals to meet the needs of individual CYP? How is this followed up/monitored?

1. Are you aware of any policies/practices in schools to help children with making healthy food and drink choices?

1. What are the CYP general understanding of what are healthy choices?

1. What do you know about the school food standards – how are they implemented - any aspects that you think are important/particularly relevant for children with additional needs?

1. How do you think schools can help children with additional needs have a healthier diet in school?
2. Is there anything else you would like to discuss about the food/drink provided and/or support with food and drink choices for CYP in school?

**The CHOICE Study**

**Head teacher/teacher interview topic guide**

**Introduction**

- Thank participant for taking part and giving up time.
- Confirm they have read the participant information sheet and ask if they have any questions about the study.
- State the discussion will take around 60 minutes and they are free to stop taking part at any time if they choose to do so.
- Confirm they are happy to go ahead with the discussion. Check they have completed the e-consent form, if not, ask for verbal consent and request the e-consent be completed post-interview.
- Inform participant that there are no right or wrong answers but that you just want to talk about their experiences of the school food and drink provision and process at their school.
- Confirm use of Zoom/Teams recording and they are happy to be recorded.
- *Remember to switch on the recorder*

**Questions**

1. We want to find out what you think about the food served at school. How well does it meet the needs of students with SEND?

1. Can you describe your school’s food and drink provision

*Prompts*

1. Caterers (private/LA)
2. Meals provided onsite or brought in from another source
3. Menu development
4. How much teacher input

1. Can you tell me about the set-up of your school for providing food and drinks for the children.

*Prompts*

1. Dining hall
2. Number of children eating at one time
3. Support staff
4. How are children supported at mealtimes
5. Different needs
6. Cultural/religious needs

1. How are parents informed about what is available for their child?

*Prompts*

1. Menus (how often) (online/printed? Do they show photos?)
2. Are parents/carers able to choose what their child will receive?
3. Do you provide feedback to parents/carers on what their child has eaten that day?
4. Other food/drinks other than lunch-time meal

1. Who else is involved in the planning/ provision of food and drink in the school?

*Prompts*

1. Parents/carers
2. School pupils
3. School councillors
4. School staff
5. Dietitian
6. Speech and language therapist

1. What policies/healthy eating practices do you use in school?

1. What do you know of the school food standards?

1. Are school food standards relevant within your school setting?

1. How are school food standards implemented in school?
2. what are the challenges, benefits etc?
3. If not being used, are there reasons why not - difficult to implement etc - not realistic, does the catering budget need to be subsidised?

1. In relation to diet, do you have any concerns about the types of food children eat at school and their health?
2. Portion sizes/amount?
3. How do you think schools can help children with additional needs have a healthier diet?
4. How does healthy eating and making healthy food and drink choices feature in the curriculum?
5. How do you think schools can work with parents/carers in encouraging healthy school food choices?
6. Is there anything else you would like to discuss about the food/drink provided and support towards healthier school food choices received at your child’s school?

Thank you for taking the time to share your experiences with me today.

I will send you some more information (debrief document) about our study, if you have any further questions, please do get in touch.

**The CHOICE Study**

**Education (teaching) staff topic guide**

**Introduction**

- Thank participants for taking part and giving up time.
- Confirm they have read the participant information sheet and ask if they have any questions about the study.
- State the discussion will take around 1 to 2 hours and they are free to stop taking part at any time if they choose to do so.
- Confirm they are happy to go ahead with the discussion. Check they have completed the e-consent form, if not, ask for verbal consent and request the e-consent be completed post-interview.
- Inform participants that there are no right or wrong answers but that you just want to talk about their experiences of the school food and drink provision and process at their child’s school.
- Remind participants to speak one at a time and to respect other people’s views if they do not agree with their own.
- Confirm use of Zoom/Teams recording and they are happy to be recorded.
- *Remember to switch on the recorder*

**Questions**

1. Please could you introduce yourself, state your professional role, and the age range of child or young person you work with.

1. We want to find out what you think about the food served at school. How well does it meet the needs of students with SEND?

1. How important is food and drink provision for children’s health and well-being?

1. Who chooses what food and drink a CYP will consume in school? When do they choose (e.g. at home weekly, in the classroom the morning of?
2. CYP
3. Parent
4. Classroom staff

1. What support with food and drink choices do CYP receive from school staff? How is this provided? Can they change their mind?

1. How does the mealtime environment in your school contribute to the food and drink choices made by pupils in school?

1. Dining hall set-up
2. Number of children eating at one time
3. Noise levels, other distractions
4. Placement
5. How are children supported at mealtimes
6. Number of portions available for that particular food choice

1. What other factors influence CYPs school food and drinks choices?

1. What should be the role of school staff in supporting CYP with food and drink choices?

1. What learning do CYP have about healthy food and drink in school?

1. Food and drinks
2. Portions

1. Do you think that the types of food and drink on offer are healthy, should these be changed in any way?
2. Amount of food and drink on offer

1. How well do the foods and drinks on offer cater for the needs of CYP with diverse eating and drinking requirements?

1. Are you aware of any policies/practices in schools to help children with making healthy food and drink choices?

1. What do you know about the school food standards – how are they implemented - any aspects that you think are important/particularly relevant for children with additional needs?

1. How do you think schools can help children with additional needs have a healthier diet in school?

1. Are you aware of any children that are hungry at school (and when)?

1. Do you provide feedback to parents/carers on what their child has eaten that day (including other drinks/snacks)
2. Is there anything else you would like to discuss about the food/drink provided and/or support with food and drink choices for CYP in school?

**The CHOICE Study**

**Catering provider interview**

**Introduction**

- Thank participant for taking part and giving up time.
- Confirm they have read the participant information sheet and ask if they have any questions about the study.
- State the discussion will take around 30-60 minutes and they are free to stop taking part at any time if they choose to do so.
- Confirm they are happy to go ahead with the discussion. Check they have completed the e-consent form, if not, ask for verbal consent and request the e-consent be completed post-interview.
- Inform participants that there are no right or wrong answers but that you just want to talk about their experiences of the school food and drink provision and process at specialist schools.
- Confirm use of Zoom/Teams recording and they are happy to be recorded.
- *Remember to switch on the recorder*

1. How well does current school food provision cater for the needs of pupils in specialist schools?
2. What information about the needs of children and young people within the school is available?
3. How is this obtained?
4. How often are there follow-up meetings?
5. Which other professionals are involved in the planning/ provision of food and drink in the school? How are they involved?

*Prompts*

1. Parents/carers
2. School pupils
3. School councillors
4. School staff e.g. key workers
5. Dietitian
6. Speech and language therapist
7. Occupational Therapist

1. What policies/healthy eating practices do you apply to specialist school food?

1. How are the school food standards applied for specialist schools?

1. Are school food standards achievable within the specialist school setting you cater for?

1. How are school food standards implemented?
2. what are the challenges, enablers, benefits etc?
3. If not being used, are there reasons why not - difficult to implement etc - not realistic

1. In relation to diet, do you have any concerns about the types of food children eat at school and their health?
2. Portion sizes?

1. What provision is there for cultural/religious differences e.g. halal?

1. How do you think caterers can help children with additional needs have a healthier diet?

1. Is there anything else you would like to discuss about the food/drink provided and support towards healthier school food choices received at your child’s school?

Thank you for taking the time to share your experiences with me today.

I will send you some more information (debrief document) about our study, if you have any further questions, please do get in touch.

**The CHOICE Study**

**Canteen staff interview topic guide**

**Introduction**

- Thank participant for taking part and giving up time.
- Confirm they have read the participant information sheet and ask if they have any questions about the study.
- State the discussion will take around 60 minutes and they are free to stop taking part at any time if they choose to do so.
- Confirm they are happy to go ahead with the discussion. Check they have completed the e-consent form, if not, ask for verbal consent and request the e-consent be completed post-interview.
- Inform participant that there are no right or wrong answers but that you just want to talk about their experiences of the school food and drink provision and process at their school.
- Confirm use of Zoom/Teams recording and they are happy to be recorded.
- *Remember to switch on the recorder*

**Questions**

1. We want to find out what you think about the food served at school. How well does it meet the needs of pupils with SEND?

1. Can you tell me about the set-up of your school for providing food and drinks for the children.

*Prompts*

1. Caterers (private/LA)
2. Meals provided onsite or brought in from another source
3. Menu development
4. Dining hall
5. Number of catering staff
6. Support staff
7. Number of children eating at one time
8. How are children supported with food and drink choices at mealtimes
9. How are you made aware of special food and drink requirements for individual children?
10. What support and guidance do you get from health professionals e.g. dietitians and speech and language therapists about specific individual requirements?
11. What is the guidance on portion size and additional helpings?
12. What factor’s influence children and young people’s school food and drink choices?

1. Who else is involved with the provision of food and drink in the school?

*Prompts*

1. Parents/carers e.g. provision of specialist meals
2. Children e.g. specific condiments
3. Do children/families bring in their own food?

1. Do you have any concerns about the types of food children eat at school and their health?
2. How do you think schools can help children with additional needs have a healthier diet?
3. How do you think schools can work with parents/carers in encouraging a healthy diet?
4. As a member of the catering staff, do you have any role in supporting healthy food and drink choices?
5. Are you aware of any policies/practices in schools to help children with making healthy food and drink choices?
6. What do you know about the school food standards – how are they implemented - any aspects that you think are important/particularly relevant for children with additional needs?
7. How are cultural/religious needs e.g. halal catered for?

1. Is there anything else you would like to discuss about the food/drink provided and/or support with food and drink choices received at your school?

Thank you for taking the time to share your experiences with me today.

I will send you some more information (debrief document) about our study, if you have any further questions, please do get in touch.

**The CHOICE Study**

**Parent focus group topic guide**

**Introduction**

- Thank participants for taking part and giving up time.
- Confirm they have read the participant information sheet and ask if they have any questions about the study.
- State the discussion will take around 1 to 2 hours and they are free to stop taking part at any time if they choose to do so.
- Confirm they are happy to go ahead with the discussion. Check they have completed the e-consent form, if not, ask for verbal consent and request the e-consent be completed post-interview.
- Inform participants that there are no right or wrong answers but that you just want to talk about their experiences of the school food and drink provision and process at their child’s school.
- Remind participants to speak one at a time and to respect other people’s views if they do not agree with their own.
- Confirm use of Zoom/Teams recording and they are happy to be recorded.
- *Remember to switch on the recorder*

**Questions**

1. Please could you introduce yourself and tell the group why you were keen to take part in this focus group.

1. We want to find out what you think about the food served at school. How well does it meet your children’s/needs of pupils with SEND?

1. Can you tell me about the mealtime environment in your child’s school? What feedback does your child give about this?

*Prompts*

1. Dining hall set-up
2. Number of children eating at one time
3. Noise levels, other distractions
4. Support staff

1. Who supports children with their eating and drinking? Whose role is it?
2. Food and drink selection
3. Portion control
4. Managing distractions
5. Support with feeding (physical or non-physical eating and drinking difficulties)
6. Encouragement to eat

1. How important is school food for your child’s health and wellbeing?

1. What information do you receive from your child’s school about what food drink is available/provided?

*Prompts*

1. Menus (how often) (online/paper?)
2. Do you get feedback on what/how much your child has eaten that day?
3. Other food/drinks other than lunch-time meal

1. What input do you have in choosing what your child eats in school?
2. Can you order in advance, or are choices made on the day?
3. How do you order? (provision for those without internet?)
4. If on the day, who makes the choice?
5. If meals are ordered in advance, who makes the choice?
6. Are the foods/drinks on offer right for your child?
7. Have you ever made specific requests relating to your child’s school food e.g. restricting second portions or puddings, requesting foods for a specialist or modified diet? What was your experience of this?

1. Do you ever send in food from home?
2. What choices do children have about what to eat?
3. What influences your child’s food and drink choices (school food choices made at home or in school)?
4. What help with food and drink choices do children receive?
5. Can children ask for additional helpings?

1. Do you think that the types of food and drink on offer are healthy, should these be changed in any way?
2. How do you think schools can help children with additional needs have a healthier diet in school?
3. Are you aware of any school healthy eating policies in your child’s school?
4. Are you aware of the use of school food standards in your child’s school?
5. How are cultural/religious needs e.g. halal catered for?
6. Is there anything else you would like to discuss about the food/drink provided and healthy eating support received at your child’s school?

Thank you for taking the time to share your experiences with me today.

We would like to send you a voucher to thank you for your time.

I’m going to send you some more information (debrief document) about our study, if you have any further questions, please do get in touch.

**CHOICE**

**Young person topic guide**

1. Please could you tell us what it is like to be in your dining hall at lunchtime?
2. How does this make you feel?

1. Do you choose what you eat and drink in school by yourself?

1. Does anyone help you to choose what you will eat and drink?
2. Who helps you?

1. When do you decide what you will eat and drink?
2. Before you get to school
3. In the morning when you are at school
4. When you are in the lunch hall

1. How do you decide what you will eat and drink in school?
2. What your friends choose
3. Taste
4. Texture
5. How hungry you are
6. Other

1. How do you know what the options are for school lunch?
2. Is there a menu? If yes, what is it like? Can everyone use it?

1. Do you always get the food you have chosen?
2. If no, why not? What happens if your choice isn’t available?
3. Does anyone feel hungry at school? If yes, when during the day and how often?

1. What happens if you want to change your food and drink choice?

1. Do you ever bring in your own food?
2. Why/why not?

1. Are there any rules about what you can or cannot eat or drink at school?
2. Do pupils have any say in what happens about school food (e.g. school menu/what happens at lunch, school food rules)

1. Can you get more than one helping of food?

1. Is there enough choice of food and drink in school for all pupils?
2. What about young people who need special foods or need their food and drink to be made in a special way?

1. Do you learn about healthy food and drink in school?
2. Do you think the food and drink in school is healthy?
3. Are portion sizes in school healthy?

1. What kinds of food do you think are healthy/good for you?
2. Is eating healthily important to you?
3. Yes/no - why?
4. How could schools help pupils to make healthier food and drink choices?

**Appendix 2:**

**Additional stakeholder quotes**

1. **Meeting and supporting YP’s individual needs**

*“When I see the kids come to the table, the portion sizes are not always- I don’t think it’s always enough for the students…I don’t often think that you maybe get enough of it, to meet the calorie needs of some pupils” (TS, school C).*

*“Yeah, we've got a lot of kids who require the halal diet. The kids are always asking, “Is that halal?” So, it’s either a yes or a no. So that's probably the only one, really. No, that’s it. Just the halal. And your vegetarians and what have you” (CS, school B).*

1. **Food choice, decision makers and provision**

*“The pupils do have a choice of breakfasts in the morning so there’s fruit, there’s bagels, so there is provision in school for breakfast” (TS, school A).*

*“I used to have to send in packets of noodles, because he [YP] likes those, and I knew he would eat them. And they [school] made them. And, you know, they [staff] were just always so supportive” (P, school A).*

1. **Dining hall environment and procedures**

*“But even just getting pupils to concentrate on eating their food, that bit can be really, really hard, especially when you’ve got… you might have 40 kids in [the dining hall] at one point, and distractions are everywhere. So that’s another challenge that you face, because you’ve still got to get everybody in the space of an hour” (TS, school C).*

1. **Communication**

*“…if they’ve ever said [school staff] they're [YP] going through a stage of not eating, they'll get in touch with me and say, "What do you think?" (P, school C).*

1. **Menu planning and communication**

*“Because some of the things he [chef] offers as a menu when you describe it to the students they’d be like… they don’t understand what that is, so we’ve got the visual menu that we can use” (TS, school, A).*

1. **Impact of SEND on YP’s food consumption**

*“We’ve got one particular young man in our class with sensory issues around noise and food, and I find that [chef] is really, really helpful. He’ll come and he’ll let the young man work in the kitchen with him and do a little bit of cooking with him and smell different foods and taste different foods and talk about it” (TS, school A).*

Sensory play was also highlighted as an important strategy to help YP work through their restrictions, however, not all the schools have the resources to provide and replenish the ingredients required:

*“Around desensitisation, but actually they've [schools] got to have the dry foods, they've got to have stocks of things to be able to use and have it replenished” (AHP).*

Parents and staff also spoke of the long journey times some YP have to get to school and home on school transport, there were concerns that if the YP did not receive enough to eat/drink in the afternoon, they would be very hungry/thirsty by the time they arrived home.

*“She's [YP] definitely putting on weight now. She's a teenager, as well, so she will struggle to make the choices on the menu or, like, one, two, and three or something, I think. They're all quite heavy midday meals” (P, school D).*

*“So I think sometimes that can be an influence that they’re [YP] only exposed at home to those certain few things that they will tolerate at the moment. I think it’s education as well because obviously I’ve had a young person say that they’ve had chocolate for breakfast or pizza for breakfast and it’s about making those conversations with home and saying, “This isn’t really acceptable for breakfast” (TS, school A).*

1. **Development of YP’s food life skills**

All the schools have an element of learning about healthy eating and cooking within their curriculum. PUPILS are taught basic food preparation skills such as making fruit kebabs, pizzas etc, going shopping for ingredients, visits from external organisations, learning where food comes from, and in one school, are invited into the school kitchen to learn about meal preparation:

*“They do a lot around food as a curriculum anyway. You know, they do cooking within the classroom, and they allow the children to explore different foods that they’ve made themselves, and then eat that” (parent, school A).*

In some schools the pressure of ensuring that all pupils were fed led to a disconnect between classroom learning about food and mealtimes and dining hall practices:

*“So it's learning within the classroom rather than the dinner hall, so the dinner hall is just, "Get your dinner. Please eat,"” (staff member, school B). “[chef] will get the food in so we can link it to history and RE and we’ve linked it to Hanukkah and Chinese New Year and things like that…” (staff member, school A).*

*“So this might be the only meal that they actually sit and eat around with other people and I think that’s really quite important as well, to be able to enjoy it from that social side as well” (staff member, school A).*

1. **School food standards, guidelines and policies**

*“I know we've got five out of five stars [hygiene rating] on the door, but I don't know if that's what you mean?” (staff member, school B).*

*“There are no different standards for the SEND students to your normal standards. There is a clause in it that says you can change items to suit additional needs. So, I guess that means…if you’ve got students who are autistic, who can’t access it, they can have sausage and chips every day. That’s how I interpret it. But, there’s nothing in there about your nutritional values to help support the immune systems or anything like that…so, I think…I agree with the standards. I think there has to be it [sic], but I think they need implementing differently, and I think they need revising again. But, I don’t know how you would ever go about changing any of that” (CM, school A).*
